# Low-Cost Open-Source Device to Measure Maximal Inspiratory and Expiratory Pressures

**DOI:** 10.3389/fphys.2021.719372

**Published:** 2021-08-26

**Authors:** Claudia Aymerich, Miguel Rodríguez-Lázaro, Gorka Solana, Ramon Farré, Jorge Otero

**Affiliations:** ^1^Unitat de Biofísica i Bioenginyeria, Facultat de Medicina i Ciències de la Salut, Universitat de Barcelona, Barcelona, Spain; ^2^Faculdade de Engenharias e Tecnologias, Universidade Save – Moçambique, Maxixe, Mozambique; ^3^Centro de Investigación Biomédica en Red de Enfermedades Respiratorias, Madrid, Spain; ^4^Institut d'Investigacions Biomediques August Pi Sunyer, Barcelona, Spain

**Keywords:** open-source hardware, measuring devices, respiratory monitoring, lung function, inspiratory and expiratory pressures, low cost devices, low and middle income countries, point-of-care

## Abstract

The measurement of maximal inspiratory (MIP) and maximal expiratory (MEP) pressures is a widely used technique to non-invasively evaluate respiratory muscle strength in clinical practice. The commercial devices that perform this test range from whole body plethysmographs to portable spirometers, both expensive and include a wide range of other respiratory tests. Given that a portable, low-cost, and specific option for MIP and MEP measuring device is not currently available in the market. A high-performance and easy-to-build prototype has been developed and the detailed technical information to easily reproduce it is freely released. A novel device is based on an Arduino microcontroller with a digital display, an integrated pressure transducer, and three-dimensional (3D) printed enclosure (total retail cost €80). The validation of the device was performed by comparison with a laboratory reference setting, and results showed accuracy within ±1%. As the device design is available according to the open-source hardware approach, measuring MIP/MEP can greatly facilitate easily available point-of-care devices for the monitoring of patients and, most important, for making this lung function measurement tool affordable to users in low- and middle-income countries.

## Introduction

Measurement of maximal inspiratory (MIP) and maximal expiratory (MEP) pressures is an easy, non-invasive, and rapid test to assess the strength of the respiratory muscles (American Thoracic Society/European Respiratory Society, 2002; Caruso et al., 2015). MIP is the maximum negative pressure that can be generated by forced inspiration. It is generated by maximum contraction of the diaphragm and intercostal muscles which tend to increase the volume of the rib cage and consequently lung volume. MEP is the maximum positive pressure that can be generated on forced expiration when the abdominal muscles push the diaphragm and the internal intercostals up, thus tending to reduce the thorax and lung volumes. This test of breathing muscles is a routine procedure in the diagnosis of certain pulmonary diseases, specifically in patients with suspected respiratory muscle weakness. Some examples of very prevalent diseases which alter MIP/MEP values are chronic obstructive pulmonary disease (COPD), neuromuscular diseases, such as multiple sclerosis, or chronic heart failure (Laghi and Tobin, 2003; Kelley and Ferreira, 2017; Nambiar et al., 2018; Laveneziana et al., 2019).

Traditionally, the MIP/MEP test has been performed in lung function labs by means of whole-body plethysmography equipment, which is very expensive (>€50,000). In recent years, several companies have invested in the development of portable solutions, mainly regarding spirometry tests. Although the cost of these portable spirometers is significantly lower than the whole-body plethysmograph equipment, the devices are still too expensive (~€2,000) for low- and middle-income countries (LMICs). Interestingly, affordable and easy-to-use open-source hardware electronics, such as Arduino, or distributed digital manufacturing strategies, such as three-dimensional (3D) printing, have become disruptive tools to design new research and medical devices in a cost-effective way without compromising the quality of the performance. Moreover, the development and commercialization of very accurate, easy-to-install, compensated, and/or amplified low-cost pressure sensors has also been a key fact for the expansion of this type of low-cost application. Accordingly, the objective of this work was to develop and test a portable, low-cost, and easy-to-build device to specifically measure MIP and MEP by using the technologies mentioned above. The aim was not to simply design a performance device but, following an open-source hardware approach (DePasse et al., 2016; Eslambolchilar and Thimbleby, 2016; Pearce, 2017), to freely release all the detailed technical information required to easily replicate the device. Hence, this new device is intended to expand the accessibility of a respiratory function test, applicable at the point of care (Beyette et al., 2011), that otherwise would require much more expensive equipment.

## Methods

The components and materials employed were chosen according to the rationale of developing a device very simple to replicate with easy-to-find components, mostly through e-commerce. The device consists of a development board with a microcontroller, a liquid crystal display (LCD) screen, a pressure transducer, a rechargeable 9 V battery block, a switch, a power supply base, and a customized enclosure produced by using any conventional 3D printer.

### Electronic Components

The board chosen for this device is the Arduino Mega 2560 due to higher memory capacity to run the developed program than other Arduino boards (256 kB of FLASH memory and 8 kB of SRAM instead of 32 and 22 kB, respectively of the well-known Arduino UNO). The selected LCD touch screen (Open Smart 3.2-inch touch screen TFT LCD Shield) is compatible with the Arduino Mega 2560 and has a resolution of 240 × 400 pixels. The use of a touch screen avoids the need for buttons or any other external component to select the parameters for the measurement. The pressure transducer employed is a piezoresistive strain gauge. Considering that MIP and MEP values range from −100 cmH_2_O to more than +140 cmH_2_O, the SSCDRNN160MDAA5 (Honeywell pressure sensor, Charlotte, NC, US), with a differential pressure range of ±163 cmH_2_O was chosen. This sensor is provided with in-factory calibration.

### Driving Code

**Figure 1:**
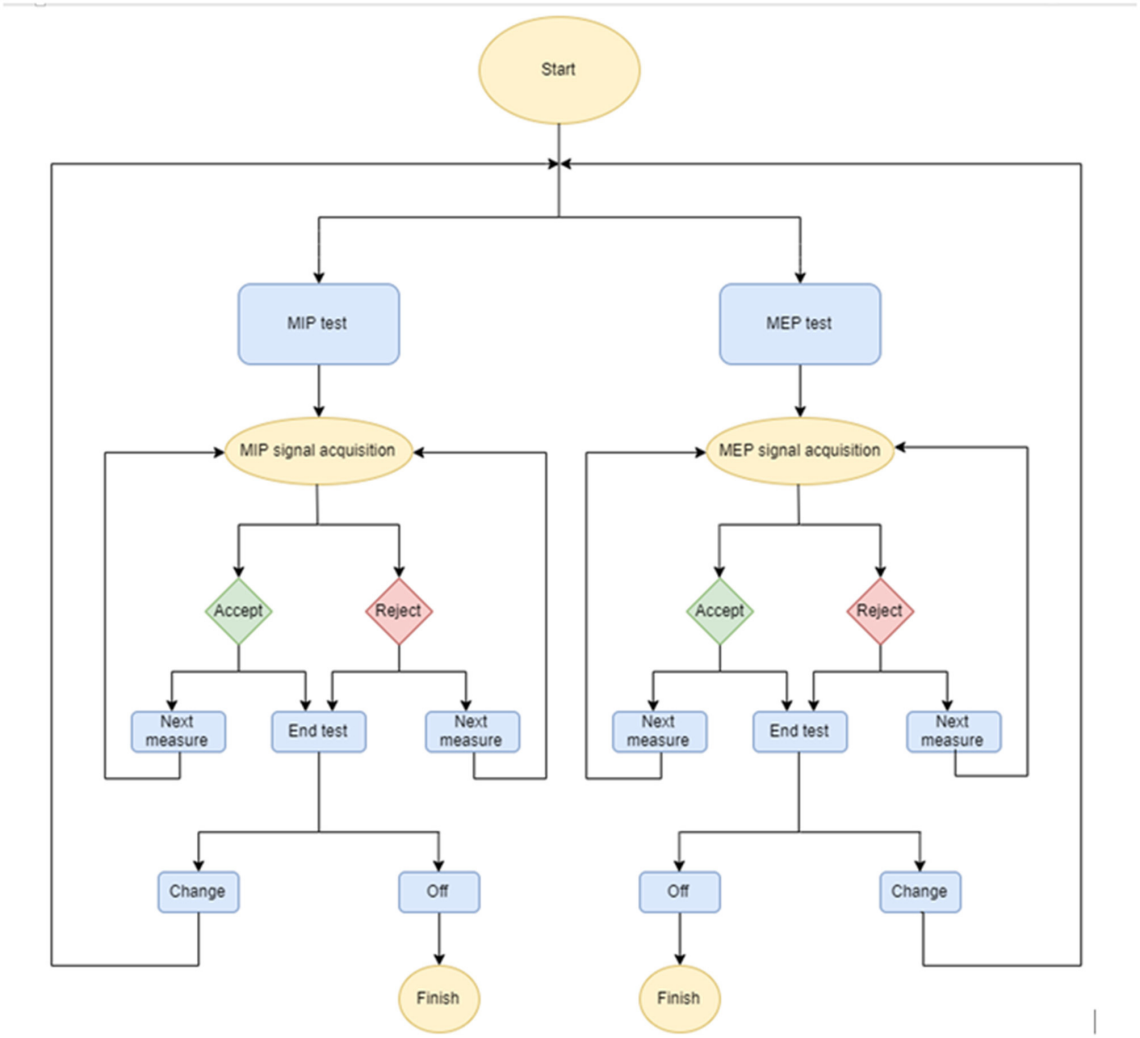
Flow diagram of the maximal inspiratory (MIP) and maximal expiratory (MEP) pressures device.

The operating code was developed with the Arduino Integrated Development Environment (Arduino IDE, Somerville, MA, US), which supports the C and C++ languages. The diagram of the developed code is shown in Figure 1. Briefly, a measuring session starts by asking the user to select running a MIP or MEP measurement, and then data acquisition starts immediately (see user manual in the Supplementary File “Technical_Description”). The acquisition lasts 5 s and is carried out with a sampling frequency of 70 Hz. After 5 s of data sampling (70 Hz), the device screen shows the corresponding pressure-time curve and the MIP/MEP value (computed as the maximum pressure sustained for 1 s) (Laveneziana et al., 2019), asking the user whether the maneuver should be accepted or not and whether a new maneuver will be carried out. After subsequent repeated maneuvers, the device shows all previously accepted maneuvers and indicates whether the quality control criterion to select the final result has been achieved: The maximum value of three maneuvers that vary by <10% (Laveneziana et al., 2019).

### Three-Dimensional Enclosure

The device is designed to have two independent blocks. The first one is the hand-held framework (to be used by the MIP/MEP test technician) containing the electronics and digital display of the measurement process and results. The second one is a hand-held mouthpiece support to contain a disposable mouthpiece for the patient. Both blocks are connected through a 1-m length (3 mm ID) silicone tube. The mouthpiece framework incorporates two small holes. One of them communicates the airway opening (mouthpiece) to the pressure transducer through the silicon tube. The other orifice allows a small air-leak moving from/to the airway to the room air, which is required to prevent the closure of the glottis during forced inspiration and to decrease use of the oral muscles during forced expiration (Laveneziana et al., 2019).

### Open Source Description

Detailed information of the circuits, electrical connections, driving code, and files for 3D printing are provided in Supplementary File “Technical_Description.zip.”

### Device Testing

The accuracy of the device was tested in a conventional way (Beyette et al., 2011) by comparing its performance with a reference laboratory setting based on a specifically calibrated and well-characterized pressure transducer. The signal of a reference transducer (Honeywell 26PC Series) was sampled with an AD/DA board and LabVIEW software, and stored for subsequently computing MIP/MEP with a Python 3 script of the same algorithm within the Arduino in the device under test. This allowed to precisely check all the measuring steps carried out by the Arduino setting in the device. For the performance test, the pressure outlet of the device mouthpiece framework was sensed simultaneously by the device sensor and the reference setting sensor. Two subjects from the technical staff who were familiar with the MIP/MEP measurements performed a series of 64 maneuvers with different intensities to mimic the ample range of values found in clinical practice. The degree of agreement between measures obtained by the designed device and reference equipment was carried out by Bland–Altman analysis. Moreover, a linear regression plot was carried out for evaluating the device vs. the reference equipment measurements.

## Results

**Figure 2:**
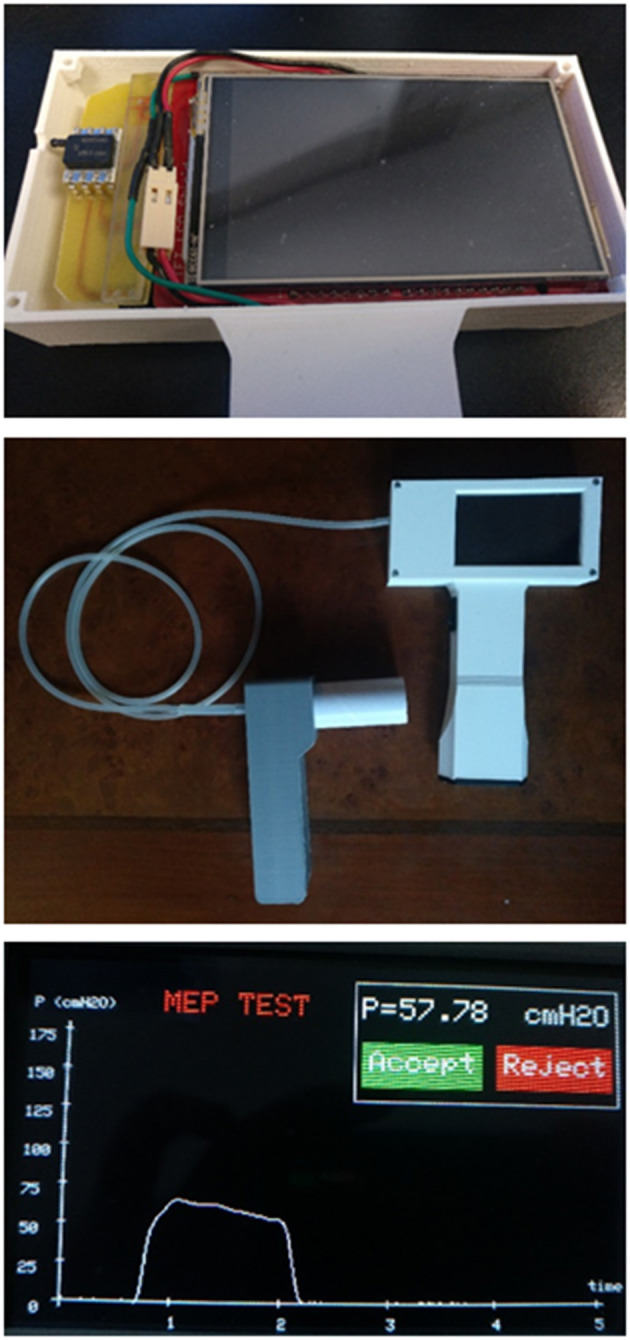
(Top) Inside view of the MIP and MEP device prototype showing the operator enclosure, the pressure transducer, and the liquid crystal display (LCD) screen [on top of the Arduino controller (not visible)]. (Center) Complete external view of the device showing the operator hand-held block and the mouthpiece block for the patient, connected through a silicone flexible tube. (Bottom) Example of one of the screens during device operation, showing the result of an MEP maneuver test (time course of expiratory pressure, MEP result, and option to allow the user to accept or reject this specific maneuver).

An image of the assembled prototype is shown in Figure 2. This figure (bottom) also shows a view of one of the screens of the device appearing during the acquisition of an MEP signal. Importantly, Table 1 summarizes the components cost of the prototype which amounted to €80.

**Table 1:** Retail cost of components used in the device.

**Component**	**Price**	**Units**	**Total**
Arduino Mega 2560	35 €	1	35 €
LCD	20 €	1	20 €
Transducer	10.36 €	1	10.36 €
PCB copper sheet	4.57 €	1	4.57 €
PLA (for 3D printer)	20 €/kg	0.25 kg	5 €
Silicone tube	0.47 €/m	1 m	0.47 €
Switch	1.29 €	1	1.29 €
Power supply base	0.83 €	1	0.83 €
Total			77.52 €

**Figure 3:**
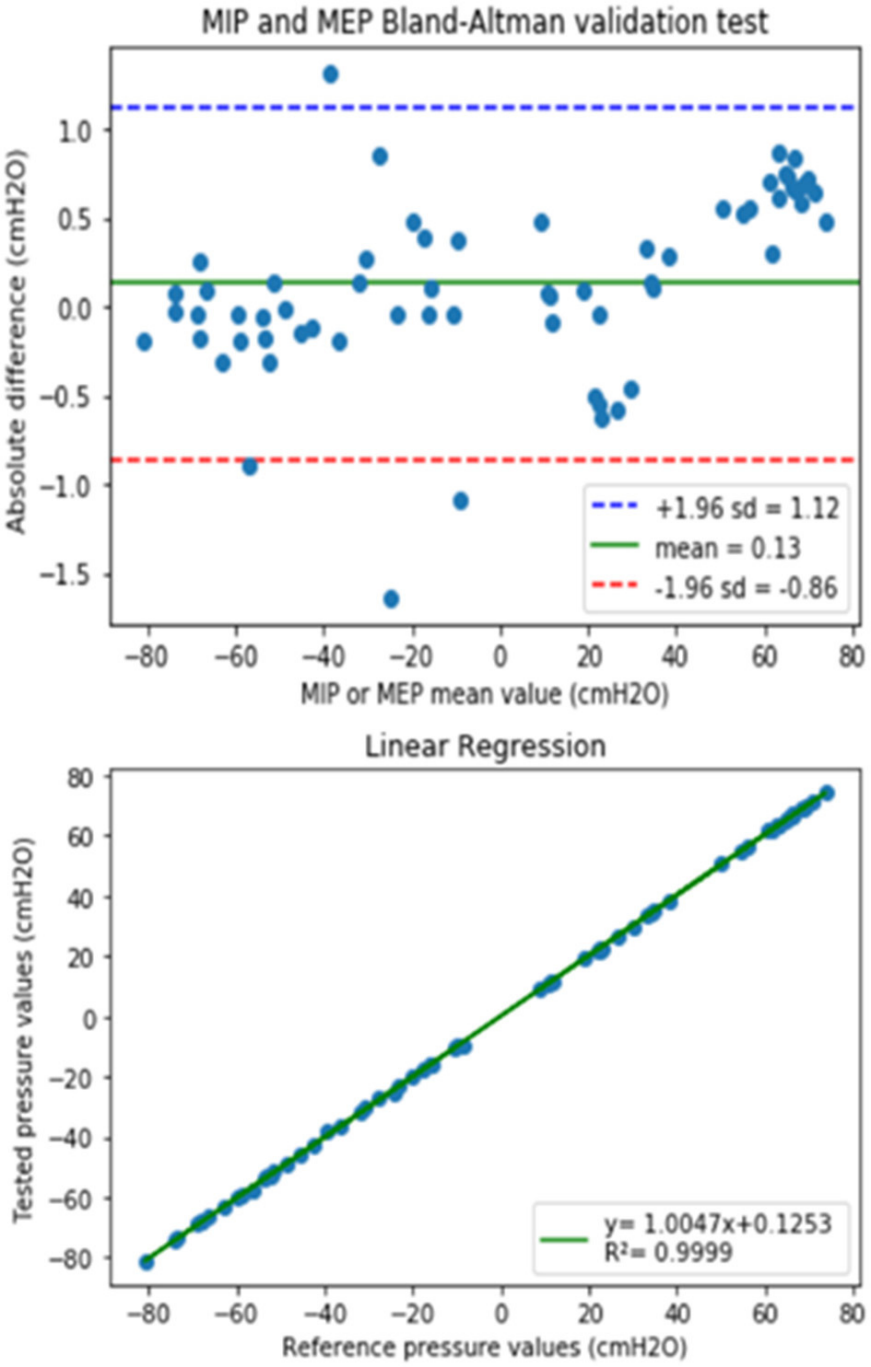
(Top) The Bland–Altman plot showing the difference between values measured by the prototype and the reference equipment, as a function of the measured values for both MIP (negative values) and MEP (positive values). Green line is the prototype bias and blue-red lines indicate the limits of agreement. (Bottom) Linear regression of the values obtained with the developed device and the laboratory reference.

The results obtained when the device was evaluated by comparison with a laboratory reference setting are presented in Figure 3 by means of the Bland–Altman (top) and linear regression (bottom) plots. The obtained average difference in MIP/MEP values from the prototype and the lab reference setting was 0.13 cmH_2_O (range of agreement from −0.86 to 1.12 cmH_2_O), which corresponds to ±1% accuracy. Therefore, the developed device is fully suitable to perform MIP and MEP measurements within clinical ranges.

## Discussion

Following the aim of this methodological work, we have designed and tested a low-cost device for measuring MIP/MEP and provided full open-source technical details allowing any interested user to directly reproduce or modify it according to the specific requirements.

The standard MIP/MEP test is aimed at non-invasively and selectively assessing the strength of inspiratory and expiratory muscles. Correct performance of this technique requires following the indications published by medical societies, such as the American Thoracic Society (ATS) and the European Respiratory Society (ERS) that agreed to establish a standard protocol, which was published in 2002 (American Thoracic Society/European Respiratory Society, 2002) and was updated by the ERS in 2019 (Laveneziana et al., 2019). In practice, MIP/MEP measurements are obtained in the seated position of patient. Maximum inspiratory (Mueller maneuver) and maximal expiratory (Valsalva maneuver) measurements should be supervised by a trained technician, who must ask the patient to exert his/her maximal effort. Both forced inspiratory and expiratory muscle efforts should ideally be maintained for at least 1.5 s, allowing that maximum pressure is measured for a 1-s period. The MIP/MEP device should give a visual feedback of the patient maneuver by displaying the pressure-time curve and the value of the 1-s average maximum pressure. After observing the pressure-time curve along the maneuver, the technician should determine whether it has been satisfactorily performed. Finally, the maximum value of three correctly performed maneuvers which vary by <10% is the retained figure (Laveneziana et al., 2019). The algorithm implemented in the designed device follows these recommendations and guides the technician along a clear and user-friendly procedure. Moreover, the low-cost device presented herein covers all the potential wide range of pressures that can be found in both healthy young people and in patients with severe dysfunction of respiratory muscles, with high accuracy (Figure 3).

The MIP/MEP measurement technique is in fact very simple from both conceptual and technical viewpoints. Indeed, it is based on recording pressures at the mouthpiece, computing the average of high pressures along a 1-s of stable inspiratory/expiratory effort, and providing the variability among values in subsequent maneuvers to select the maximum value among several representative muscle efforts. The design and construction of the device illustrate how fruitful could be a multidisciplinary approach. In fact, common projects carried out following a collaborative scheme have already produced several examples of low-cost open-source devices for both research and treatment (Farré et al., 2019a,b; Garmendia et al., 2020; Osuna et al., 2021).

The novelty of the device described here is that it is low-cost and easy-to-build from fully disclosed technical information. Indeed, other simple devices with a similar function were described but the technical details allowing their simple replication by other potential users were not provided (Smith and Royall, 1992; Hamnegård et al., 1994; Maruthy and Vaz, 1999; Torres-Castro et al., 2019). Accordingly, this device is of interest for two potential application scenarios. On the one hand, it may facilitate the affordable provision of a considerable number of devices to be used as point-of-care tools (Pearce, 2012). Indeed, MIP/MEP measurements have potential interest for monitoring respiratory muscle strength as a biomarker of progress/recovery in extremely prevalent diseases, such as COPD and heart failure. Having affordable MIP/MEP devices available for extended home monitoring of patients may allow for carrying out clinical studies that otherwise would not be possible. On the other hand, the device described in this work opens the opportunity to provide a low-cost tool to patients and doctors in low- and middle-income countries (LMICs). In this regard, it is interesting to mention that the low-tech components required to build the device make it possible that its construction and maintenance are performed by teams of engineers in LMICs (De Maria et al., 2014; Mackintosh et al., 2018). It is also noteworthy that the collaborative approach followed in this study, consisting of co-creation and design thinking (Ranger and Mantzavinou, 2018) by teams in Mozambique and Barcelona may help toward moving the design focus from the developed country perspective to that of the LMIC team and to potentially stimulate the development of local industry (Clifford and Zaman, 2016).

## Data Availability Statement

The original contributions presented in the study are included in the article/Supplementary Material, further inquiries can be directed to the corresponding author/s.

## Author Contributions

JO and RF: conception and supervision of the study. MR-L, CA, and GS: experimental design and implementation. CA, MR-L, and RF: draft preparation. RF and JO: manuscript edition. All authors final manuscript review and approval.

## Conflict of Interest

The authors declare that the research was conducted in the absence of any commercial or financial relationships that could be construed as a potential conflict of interest.

## Publisher's Note

All claims expressed in this article are solely those of the authors and do not necessarily represent those of their affiliated organizations, or those of the publisher, the editors and the reviewers. Any product that may be evaluated in this article, or claim that may be made by its manufacturer, is not guaranteed or endorsed by the publisher.
