# Supplementary material for: Low-Cost Open-Source Device to Measure Maximal Inspiratory and Expiratory Pressures
Source: Front Physiol. 2021 Aug 26;12:719372. doi: 10.3389/fphys.2021.719372 (PMC8427661; doi:10.3389/fphys.2021.719372)
Supplement: Supplementary file 1 [file Data_Sheet_1.ZIP › Technical_Description/Technical_Data_and_User_Manual.pdf]

OPEN SOURCE HARDWARE PUBLICATION

TECHNICAL DESCRIPTION

LOW-COST OPEN-SOURCE DEVICE TO MEASURE MAXIMAL INSPIRATORY AND EXPIRATORY PRESSURES

Claudia Aymerich[1], Miguel Rodríguez-Lázaro[1], Gorka Solana[2], Ramon Farré[1,3,4], Jorge Otero[1,3, *]

[1] Unitat de Biofísica i Bioenginyeria, Facultat de Medicina i Ciències de la Salut, Universitat de Barcelona, Barcelona, Spain.

[2] Faculdade de Engenharias e Tecnologias, Universidade Save - Moçambique

[3] CIBER de Enfermedades Respiratorias, Madrid, Spain

[4] Institut d'Investigacions Biomediques August Pi Sunyer, Barcelona, Spain

*Corresponding author:
Dr. Jorge Otero
Unitat de Biofísica i Bioenginyeria
Facultat de Medicina i Ciències de la Salut
Casanova 143, 08028 Barcelona, Spain
Email: jorge.otero@ub.edu

DISCLAIMER

This document provides technical information on the specific device prototype described in the publication indicated above.

Using components different from (although similar to) the ones described here may require technical adjustments or adaptations and therefore the final performance of the resulting device must be specifically assessed.

The authors of this document are not responsible for the use of the information contained herein nor for any device built using such information.

LICENSE:

This device is released under CERN Open Hardware License (OHL) v1.2 and all software and documentation under GNU General Public License (GPL) v3.0.

Content:

1. *Device code*
2. *Schematics of the circuit*
3. *PCB board layout*
4. *Connections*
5. *Enclosure by 3D printer*
6. *User manual*

1. Device code

Source code in file: CODE_Arduino.ino (included in the same zip folder where this pdf file is located).

2. Schematics of the circuit

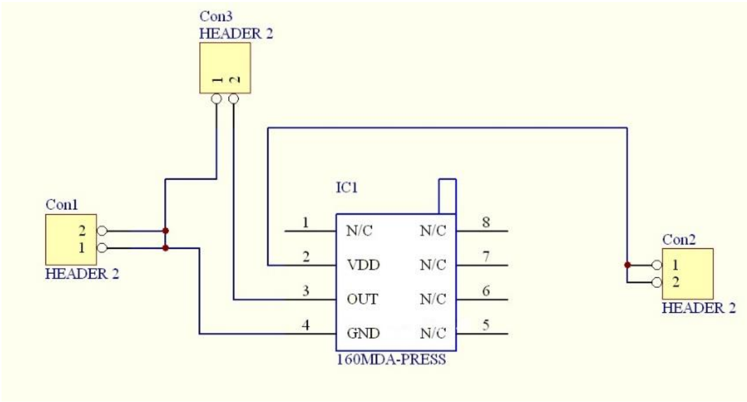

3. PCB board layout

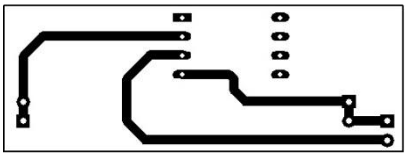

Actual size: 53x19 mm

4. Connections

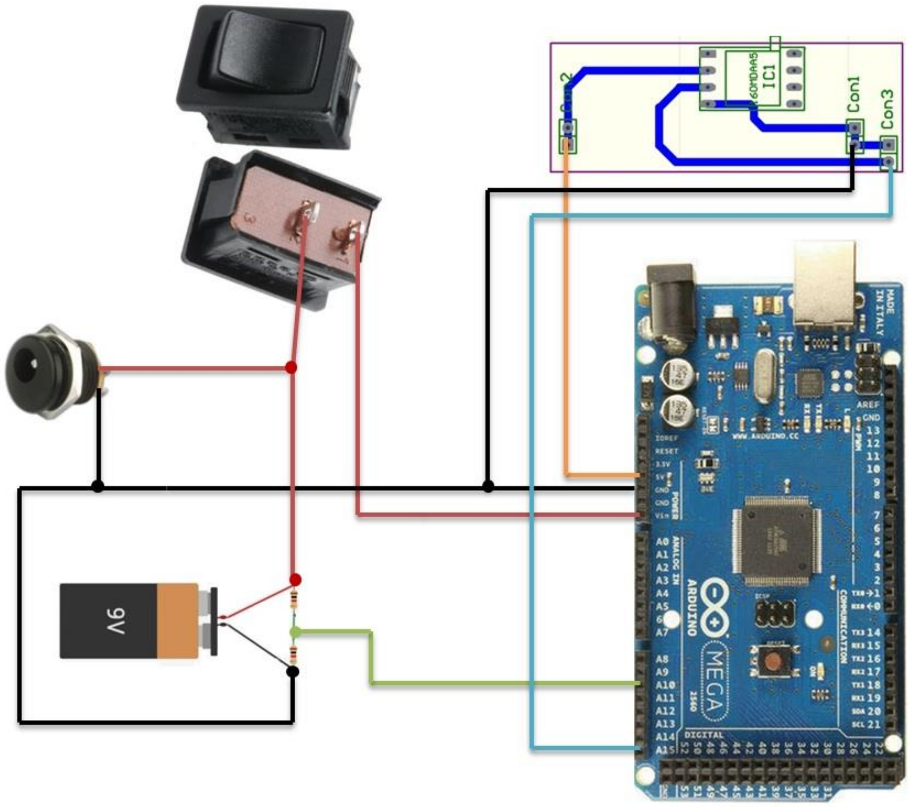

5. Enclosure by 3D printing

Source code in files: 3D-file1.stl, 3D-file2.stl, 3D-file3.stl (included in the same zip folder where this pdf file is located).

- Framework

It consists on a rectangular block, adapted to the dimensions of the Arduino, LCD shield and the PCB, united to the handle block and a cover. The rectangular block contains some slots for ventilation to avoid the Arduino board overheating, 4 supports for the screws, 1 support to avoid the Arduino to move, a small slot for the connection of the transducer to the silicon tube and a hole for the USB type B port of the Arduino. To optimize the device, it was determined to place the battery in the handle. The 9V block and its support had perfect dimensions to adapt to this idea. In this way, the battery could easily be removed from the bottom of the handle. The handle contains a hole, as well, to place the power switch and another one to place the supply base. Finally, the cover contains the holes for the screws, the transducer slot and a rectangular hole adapted to the dimensions of the visible part of the LCD.

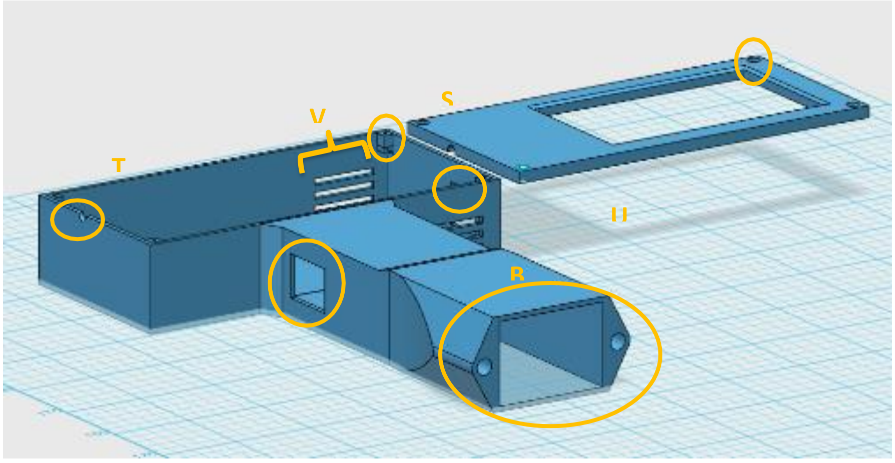

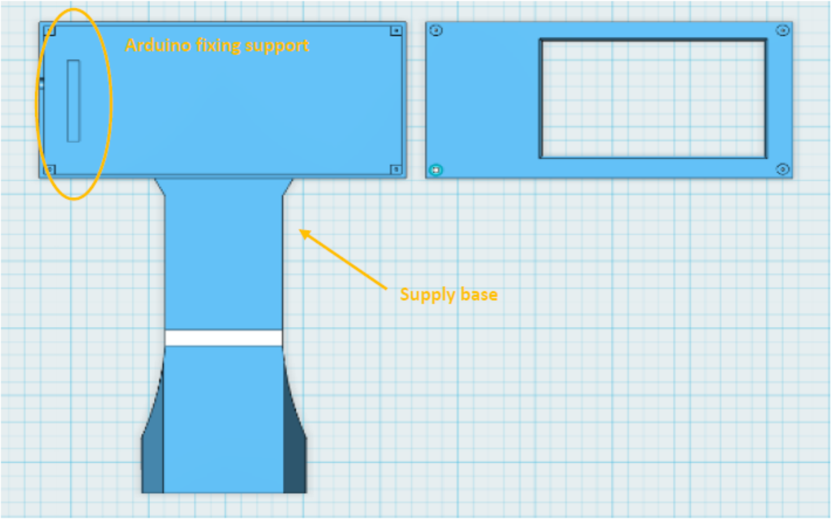

- Mouthpiece support

It consists of two jointed blocks of different depth. The first block consists on the handle and the second block is adapted to contain the disposable carton mouthpiece. Two small holes have been incorporated. The one in the centre connects the air in the mouthpiece to the transducer along the silicon tube. The second one, on its right, consist on a small loss of air. It is required to prevent closure of the glottis during forced inspiration and decreased use of the oral muscles during forced expiration.

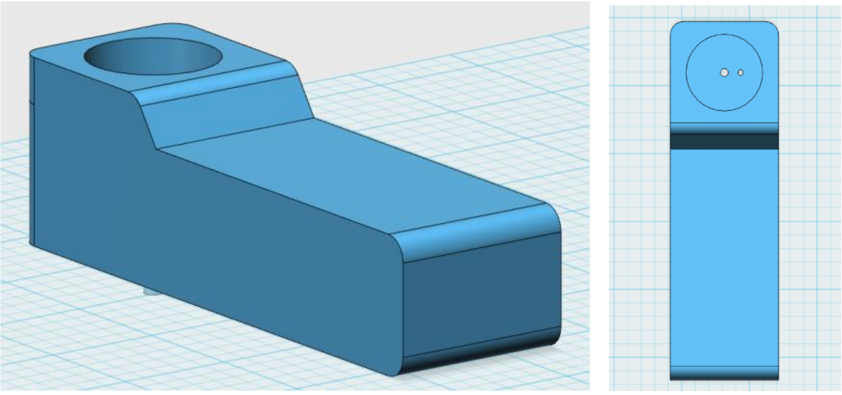

6. User manual

DEVICE TO MEASURE

MAXIMAL INSPIRATORY AND EXPIRATORY PRESSURES (MIP / MEP)

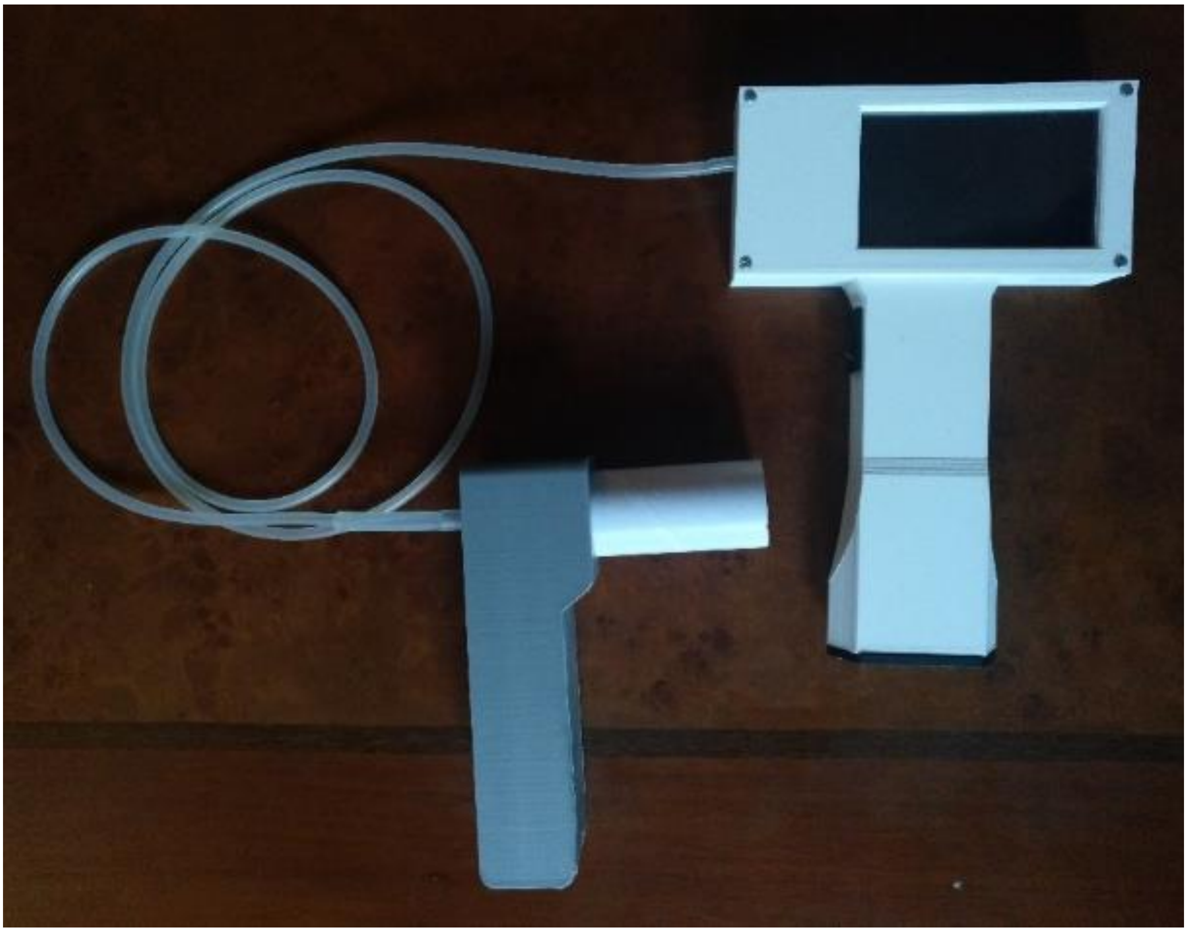

0. Prior instructions

Place a disposable mouthpiece on the corresponding holder and give it to the patient.

1. Home screen

Press the switch to start the device. Then, press the button of the test you want to perform. Options to choose from:

-MIP test (Measurement of maximum inspiratory pressure)

-MEP test (Measurement of maximum expiratory pressure)

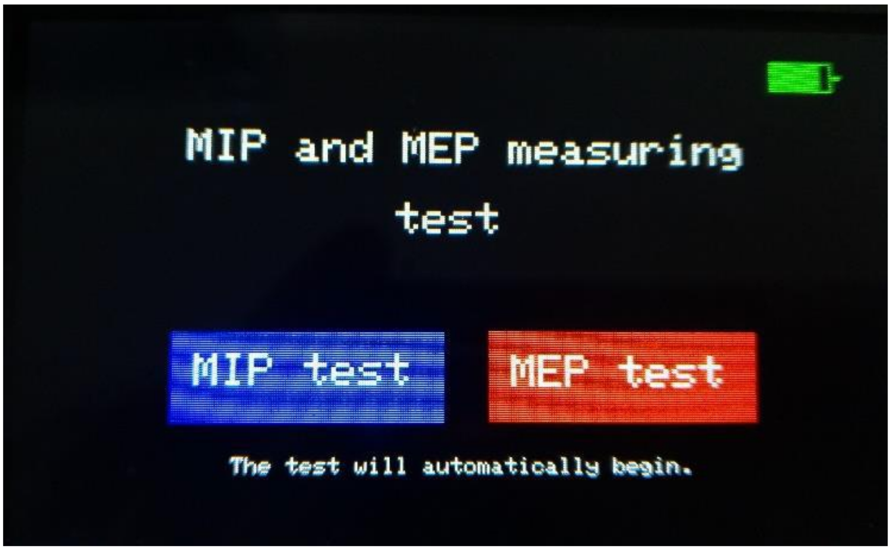

Figure 1. Home screen

If you choose a wrong button, you will not be able to change the test until step 3.

2. Carrying out the test

Have the patient perform the maximum inspiration (or expiration) maneuver. It must last at least 1 second. The maximum time to perform the test is 5 seconds. The screen will display the maximum inspiratory (or expiratory) pressure value of the measurement performed (Figure 2).

If the maneuver has been done correctly, you should observe a curve similar to that in Figure 3. In this case, press the "Accept" button. The measurement will be saved in the device memory. To reject the measurement, press the "Reject" button.

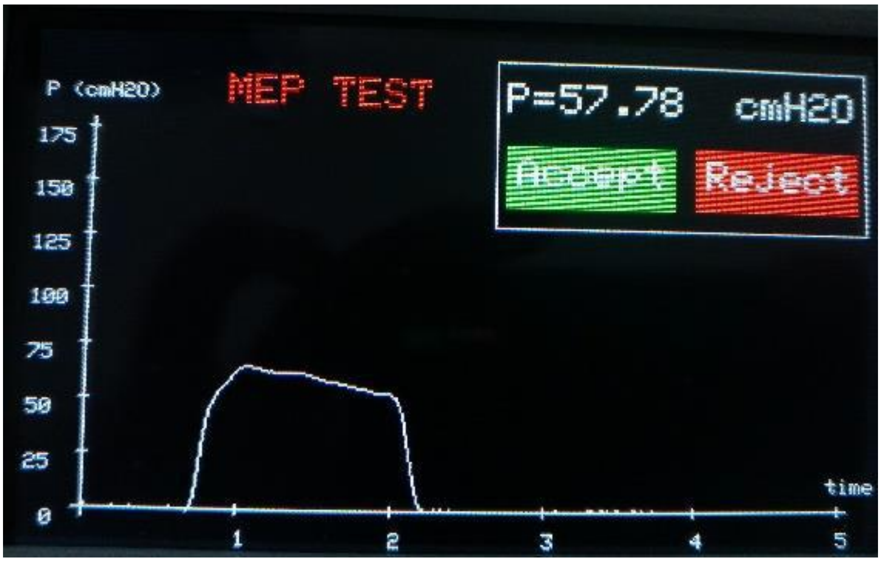

Figure 2. Test run

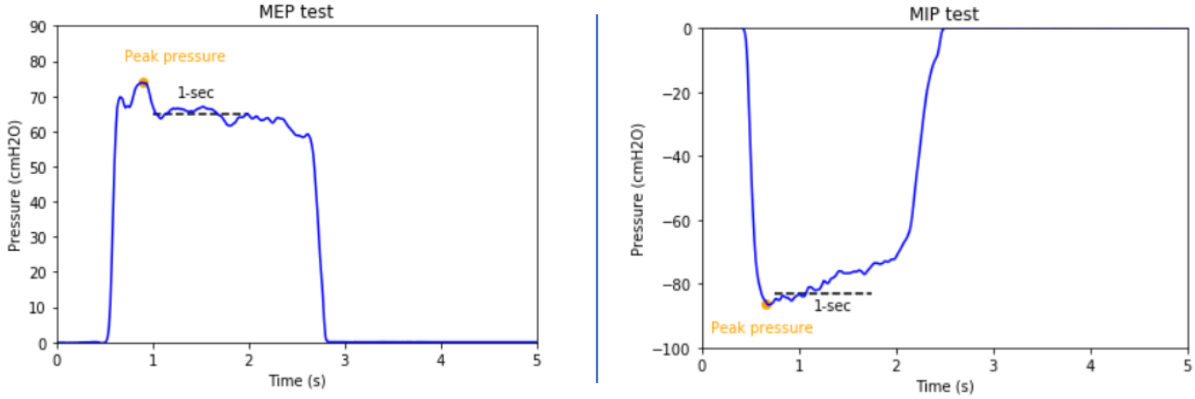

Figure 3. Illustration of correct MEP (Pemax) and MIP (Pimax) measurements

3. Repetition of the test

To perform the next test, press the “Next measure” button and repeat the procedure in point 2. You will see that, as you perform valid tests, the pressure values of the previous measurements will appear on the right side of the screen.

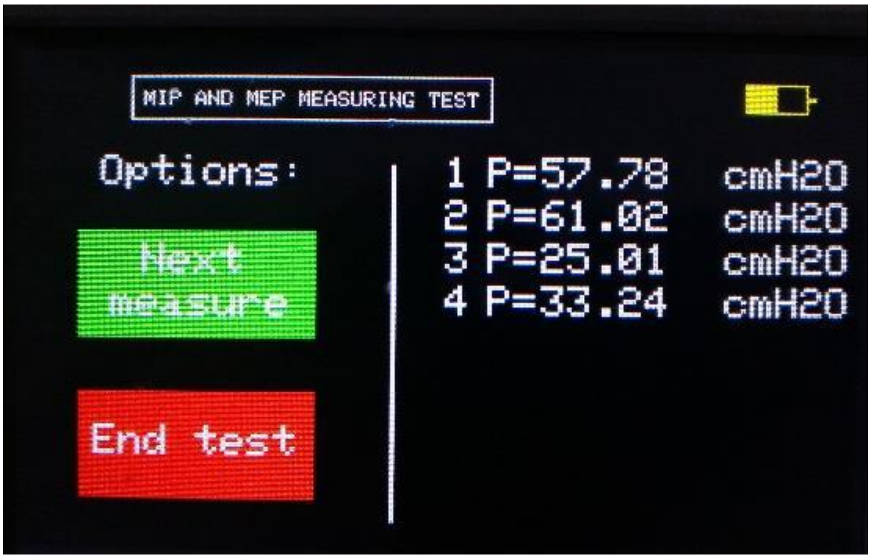

Figure 4. Screen at the end of a measurement

If you want to change the experiment or end the experiment, press the "End test" button. This button will direct you to step 5.

4. Completion of the MIP / MEP test

In the case of performing three measurements that differ by less than 10%, the device will indicate the value of MIP or MEP of that patient, as shown in Figure 5. In addition, at the bottom- on the right side of the screen you will see the three pressure values used to calculate the MIP / MEP.

The measure is terminated. The oprator can then record this value into the patient's medical record. Press the "End test" button to exit the screen. This button will direct you to step 5.

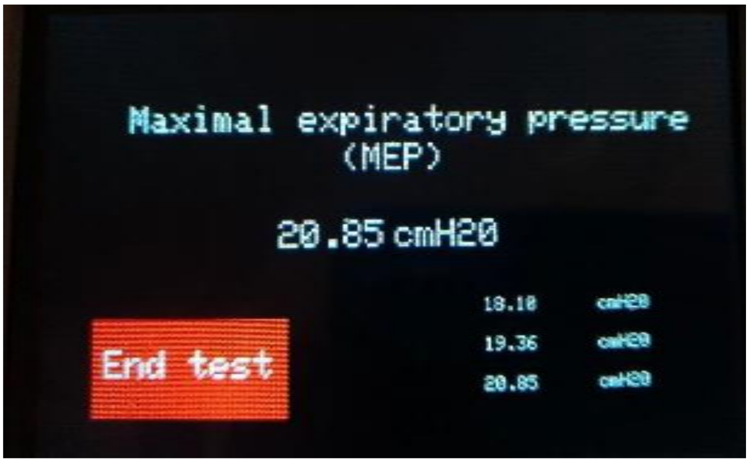

Figure 5. Example of MEP value

If you do not get three measures that differ by less than 10%, if the oprator deems it appropriate, he/she can end the test without the device telling you what the patient's MIP or MEP is. The specialist can take note of the pressure values generated in each of the valid measures to introduce them into the patient's medical history. These values are on the right of the screen, in the form of a list (see Figure 4).

Finally, select the "End test" button.

5. Test change and / or device shutdown

If you want to change the experiment, press the "Change" button. The initial screen will appear again, in which you can choose the test to be performed (MIP or MEP).

If you want to finish the test for good, press the "Off" button. The following message will appear: "Thank you". Finally press the power switch to turn off the device.

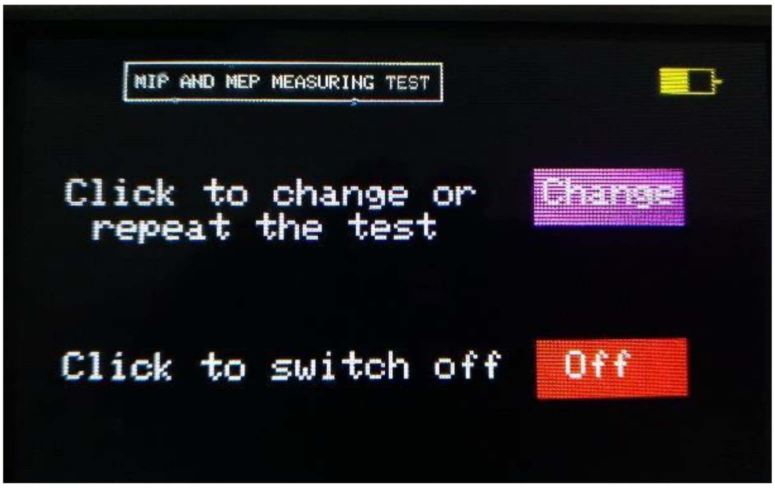

Figure 6. Test change and device shutdown

6. Battery

The battery status of the device is indicated at the top-right of the screen, on all screens except the measurement (graphic) screen. Battery status is divided into five levels:

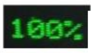

The battery is in its maximum state of charge.

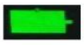

The battery has more than 50% charge

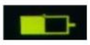

The battery is between 25% and 50% charge

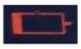

The battery is at less than 25% charge

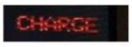

In this state, the message “Charge” appears on the screen. The device will take a few minutes to turn off.

It is recommended to charge the device if the battery icon is yellow to ensure that you have enough battery power for the next measurement, as the maximum battery life, when fully charged, is one hour. The charger comes bundled with the device, along with a time controller. It is important that the charger is connected to the time controller (which comes fixed to the base for 2 hours of charging) as otherwise the device could be damaged.
